# Cooking with biomass fuel and cardiovascular disease: a cross-sectional study among rural villagers in Phitsanulok, Thailand

**DOI:** 10.12688/f1000research.23457.2

**Published:** 2020-10-08

**Authors:** Chudchawal Juntarawijit, Yuwayong Juntarawijit

**Affiliations:** 1 Department of Natural Resources and Environment, Faculty of Agriculture, Natural Resources, and Environment, Naresuan University, Phitsanulok, 65000, Thailand; 2 Faculty of Nursing, Naresuan University, Phitsanulok, 65000, Thailand

**Keywords:** Biomass fuel, cardiovascular diseases, household air pollution, kitchen smoke, cooking fume

## Abstract

**Background: **Burning biomass fuel is a major source of indoor air pollution; about 40% of Thai people still use biomass for cooking. There is increasing evidence of the association between biomass smoke exposure and serious health effects including cardiovascular disease. The object of this cross-sectional study was to investigate the association between biomass use for household cooking and cardiovascular outcome, including coronary heart disease, hypertension, high cholesterol, diabetes mellitus, and stroke among rural villagers in Phitsanulok, Thailand.

**Methods: **Data from 1078 households were collected using a face-to-face interview questionnaire. In each household, data on cardiovascular disease, cooking practices, and cooking fuel, types of fuel they normally used for cooking, were collected.

**Results: **After being adjusted for gender, age, cigarette smoke, secondhand smoke, and exposure to other sources of air pollution, it was found that the family members of cooks using biomass fuel were at risk of coronary heart disease (CHD; OR=4.35; 95%CI 0.10–18.97), hypertension (OR=1.61; 95%CI 1.10–2.35), high cholesterol (HC; OR=2.74; 95%CI 1.66–4.53), and diabetes (OR=1.88; 95%CI 1.03–3.46). Compared to LPG use, using wood was associated with stroke (OR=7.64; 95%CI 1.18–49.61), and using charcoal was associated with HC (OR=1.52; 95%CI 1.04–2.24). Compared to never user, household cooks who sometimes use charcoal had an increased risk of hypertension (OR=2.04; 95%CI 1.32–3.15), HC (OR=2.61; 95%CI 1.63–4.18), and diabetes (OR=2.09; 95%CI 1.17–3.73); and cooks who often use charcoal had an elevated risk of stroke (OR=3.17; 95%CI 1.04–9.71), and HC (OR=1.52; 95%CI 1.02–2.27) to their family members.

**Conclusions: **The study results were consistent with those found in studies from other parts of the world, and supports that exposure to biomass smoke increase cardiovascular diseases. The issue should receive more attention, and promotion of clean fuel use is a prominent action.

## Introduction

Cooking smoke is a major source of household air pollution, which affects billions of people around the world, especially in developing countries. Globally, nearly 3 billion people still use solid fuels (wood, charcoal, crop residues, and dung) for cooking and heating ^1^. Smoke from wood burning contains a large number of pollutants, including particulate matter, carbon monoxide, nitrogen dioxide, formaldehyde, and a number of highly toxic organic compounds, such as benzene, 1, 3 butadiene, benzo[a]pyrene and other toxic polycyclic aromatic hydrocarbons ^2^. In addition to fuel burning smoke, overheated of cooking oils might also produce smoke which depended on several factors, including cooking oils, cooking methods, cooking equipment, and food types ^3^

The use of solid fuel for cooking and/or household energy sources increases respiratory and non-respiratory illnesses in both adults and children. Those effects that are well established are acute respiratory infections, chronic obstructive pulmonary disease (COPD), lung cancer, asthma, tuberculosis, and cataracts ^4, 5^. In children, biomass use is related to mortality, and acute lower respiratory tract infections, and some other non-respiratory illness, such as poor lung function, low birthweight, nutritional deficiency, and impairment of learning ability ^5, 6^.

Though with limited evidence, recent studies linked biomass smoke exposure and cardiovascular diseases (CVD), e.g. coronary heart disease (CHD), hypertension, diabetes, and stroke ^8– 11^. In laboratory studies, chronic exposure to biomass smoke increased the thickness and plaque of blood vessels ^11^. In epidemiological studies, Peruvians who live in high altitude environments and use biomass fuel had an elevated prevalence of hypertension ^13^. A study among villager women in Bangladesh reported an association between elevated cumulative exposure to biomass smoke and the prevalence of hypertension ^14^. A similar result was found in a study in Shanghai Putuo, which found using solid fuel increases the risk of hypertension, CHD, and diabetes ^15^; and a study in Shanxi, China reported an increased risk of hypertension, CHD, stroke, diabetes, and dyslipidemia ^16^. A recent study by Yu *et al*. ^17^ also linked solid fuel use to cardiovascular mortality.

On a global scale, CVD is the number one cause of death and is responsible for about 18 million deaths annually ^18^. In Thailand, CVD accounts for 23% of the national mortality ^19^. Currently, there is no study on the effect of biomass smoke on CVD in Thailand. It was reported that about 40% of Thai households still use biomass, mainly charcoal, wood, and agriculture residue, for cooking ^20^. The objective of this study is to investigate a possible association between biomass use for cooking and cardiovascular diseases, including CHD, hypertension, HC, diabetes, and stroke. The study uses data from a cross-sectional survey among rural villagers in Phitsanulok, Thailand. The result could be used for disease prevention and control, and to support the global literature.

## Methods

### Study design and setting

This is cross-sectional study. Participants are rural villagers living in Phitsanulok Province, Thailand. Phitsanulok is a midsize province located about 400 km north of Bangkok. There are 866,891 people in the area of 9 districts. Most of the people are rice farmers ^21^.

### Study participants and sampling procedure

Participants were randomly selected using multistage sampling. Out of the 9 districts in Phitsanulok province, 5 were randomly selected. In each district, one sub-district and a local health-promoting hospital were approached. In each sub-district with support from the local health-promoting hospital, a total of 1,150 households were approached and 1,134 (98.6%) people agreed to participate in the study. In each household, only one participant who was responsible for household cooking and aged over 20 years was selected. After data cleanup, 56 (4.9%) items of data were missing important information, such as age, gender, cooking practice. The final data from 1,078 people were used for statistical analysis.

The minimum sample size was calculated to be 1,034, using unmatched cross-sectional study with the following assumptions: two-sided significance level = 95%; power of detection = 80%; percent unexposed with outcome = 5%; and odds ratio = 2.0.

### Study questionnaire

Data was collected using a face-to-face interview questionnaire, which was administered by 15 village health volunteers (provided as *Extended data* in English ^22^). The interviews took place in the house of participants. The data was collected during the period of May–June 2017. Health volunteers were all trained on how to properly carry out the interview and use the questionnaire. The questionnaire was designed to collect information on demographic data, fuel use for cooking, and other cooking practices. In addition to general demographic data, participants were also asked a history of tobacco use (ever, never), and working in factory environments using “yes” or “no” questions. Ever smoker referred to those who smoke more than 100 cigarettes in their lifetime. Data on pesticide use was also measured by “yes” or “no” questions: “Have you ever spray or mix pesticide?”. For cooking fuel data, we asked about the types of fuel they used for cooking food (wood, charcoal, LPG, electricity), and the frequency of using each types of fuel. Data collected on cooking practices were types of cooking oil, the frequency of tears while cooking (TWC) (never, sometimes, often), kitchen location (inside a house, outside a house, both inside and outside a house), and the characteristics of kitchen ventilation (good or poor ventilation).

The presence of cardiovascular disease was determined by the participant response to the question: “Have you ever been diagnosed with the following diseases (coronary heart disease (CHD), hypertension, high cholesterol (HC), diabetes mellitus, stroke) by a medical doctor?”. For diseases among their family members, we asked “Did you have a family member with the following diseases?”.

The content validity of the questions was tested by three experts, and the Index of Item Objective Congruence (IOC) was between 0.7–1.0. The questionnaire was also tested for question sequencing and understanding using a group of 30 people with a similar background to the intended participants.

### Statistical analysis

Demographic and prevalence of cardiovascular disease were descriptively analyzed. Comparison between groups were analyzed using chi-square test for categorical variables, and independent t-test for continuous variables. The association between cardiovascular disease was analyzed using binary multiple logistic regression with odds ratios (OR) and 95 percent confidence interval (CI) adjusted for gender (male, female), age (continuous data), cigarette smoking (ever, never), living smoker (yes, no), working with smokers (yes, no), and exposure to air pollution (yes, no). These adjusted variables of the repondents were used also when analysis for ORs of disease risk among the respondents’ family members. All statistical analyses were performed using IBM SPSS version 19 and OpenEpi (online version 3.01). Statistical significance was set at a p-value of less than 0.05.

### Ethical considerations

The study was approved by the Ethical Committee of Naresuan University (COA No. 485/2016), and written informed consent from the respondents was obtained before the interviews were conducted.

## Results

Most of the respondents were women (84.2%) with a mean age of 53.04 ± 12.93 yr. The highest education levels were primary school or high school. Most were farmers (36.0%) and 20.2% were causal workers on farms. About 10% were smokers and 33% lived with a smoker. Additional information on the demographic data is shown in Table 1 and in *Underlying data* ^23^.

**Table 1.** Demographic data.

Characteristics (N=1078)	N (%)
**Gender**	
Male	170 (15.8)
Female	908 (84.2)
**Age (yr.)**	
20–30	67 (6.2)
31–40	136 (12.6)
41–50	205 (19.0)
51–60	343 (31.8)
61–70	258 (23.9)
71–80	69 (6.4)
Mean = 53.04 ± 12.93 (Age range 20–80 yr.)	
**Education completed**	
Primary school	757 (71.9)
Secondary school	246 (23.4)
College diploma	50 (4.8)
Missing	25 (2.3)
**Occupation**	
Farmer	388 (36.0)
Grocer	89 (8.3)
Private or government employee	57 (5.3)
Causal worker	218 (20.2)
Housewife	223 (20.7)
Other/unemployed	103 (9.6)
**Cigarette smoking**	
Ever smoke	111 (10.3)
Never smoke	967 (89.7)
**Living with smokers**	
Yes	362 (33.8)
No	710 (66.2)
Missing	6 (0.6)
**Working with smokers**	
Yes	172 (16.1)
No	895 (83.9)
**Working in a factory**	
Yes	175 (16.4)
No	894 (83.6)
Missing	9 (0.8)
**Spray or mix pesticides**	
Yes	425 (39.5)
No	651 (60.5)
Missing	4 (0.4)
**Fuel use for cooking**	
Wood	27 (2.5)
Charcoal	348 (32.3)
LPG	695 (64.5)
Electricity	8 (0.7)
**Frequency of using charcoal**	
Never	495 (46.4)
1–2 times per week	160 (15.0)
3 times per week or more	411 (38.6)
**Kitchen location**	
Inside a house	570 (53.4)
Both inside and outside	134 (12.6)
Outside a house	363 (34.0)
**Tears while cooking**	
Often	49 (4.6)
Sometimes	537 (50.8)
Never	472 (44.6)
Missing	20 (1.9)
**Cooking frequency**	
Everyday	984 (91.3)
Somedays	94 (8.7)
**Using charcoal duration (year)**	
Not use	502 (46.6)
1–20	146 (13.6)
21 or more	429 (39.8)

About 70% of the respondents reported using biomass for cooking ( Table 2). However, when asked for fuel types that they usually use for cooking, 64.5% reported LPG and 32.3% charcoal. Among those who use charcoal, 38.6% use it often. About half have a kitchen located inside a house with good ventilation. Almost all reported having TWC either sometimes or often. Most of them cook every day.

**Table 2.** Demographic data among biomass and LPG users.

Characteristics	Biomass, n (%)	LPG, n (%)	P–value *
**Gender**			
Male	114 (15.1)	56 (17.4)	0.341
Female	642 (84.9)	266 (82.6)	
**Age**			
20–30	34 (4.5)	33 (10.2)	<0.001 **
31–40	89 (11.8)	47 (14.6)	
41–50	132 (17.5)	73 (22.7)	
51–60	249 (32.9)	94 (29.2)	
61–70	204 (27.0)	54 (16.8)	
71–80	48 (6.3)	21 (6.5)	
**Education** **completed**			<0.001 **
Primary school	569 (76.9)	188 (60.1)	
Secondary school	142 (19.2)	104 (33.2)	
College diploma or higher	29 (3.9)	21 (6.7)	
Missing	16 (2.1)	9 (2.8)	
**Occupation**			<0.001 **
Farmer	299 (39.6)	89 (27.6)	
Grocer	54 (7.1)	35 (10.9)	
Private or government employee	36 (4.8)	21 (6.5)	
Causal worker	130 (17.2)	88 (27.3)	
Housewife	161 (21.3)	62 (19.3)	
other	76 (10.1)	27 (8.4)	
**Cigarette** **smoking**			0.490
Ever smoke	81 (10.7)	30 (9.3)	
Never smoke	675 (89.3)	292 (90.7)	
**Living with** **smokers**			0.475
Yes	259 (34.4)	103 (32.2)	
No	493 (65.6)	217 (67.8)	
Missing	4 (0.5)	2 (0.6)	
**Working with** **smokers**			**0.054**
Yes	131 (17.5)	41 (12.8)	
No	616 (82.5)	279 (87.2)	
Missing	9 (1.2)	2 (0.6)	
**Working in a** **factory**			**0.077**
Yes	113 (15.1)	62 (19.4)	
No	637 (84.9)	257 (80.6)	
Missing	6 (0.8)	3 (0.9)	
**Using pesticides**			**0.001** **
Yes	321 (42.6)	104 (32.3)	
No	433 (57.4)	218 (67.7)	
Missing	2 (0.3)		
**Kitchen location**			**<0.001** **
Inside a house	364 (48.6)	206 (64.8)	
Both inside and outside	109 (14.6)	25 (7.9)	
Outside a house	276 (36.8)	87 (27.4)	
**Kitchen** **ventilation**			0.580
Good	504 (96.9)	247 (97.6)	
Poor	16 (3.1)	6 (2.4)	
**Cooking** **frequency**			0.035 **
Everyday	699 (92.5)	285 (88.5)	
Someday	57 (7.5)	37 (11.5)	

* P–value of chi square test for difference between biomass user and not use group, 2–trailed test ** Significantly difference, p <0.05

The study found hypertension, HC, and diabetes to be the most common cardiovascular outcomes ( Table 3). Compared to non-user group, biomass users had a significantly higher prevalence of hypertension, and HC, and their family members also had more incidence of hypertension, HC, diabetes, and heart disease.

**Table 3.** Prevalence of cardiovascular diseases among biomass and LPG users.

Disease	Biomass, n/total n (%)	LPG, n/total n (%)	p–value *
Hypertension(R) ^a^	214/750 (28.5)	66/321 (20.6)	0.007 **
Hypertension(F) ^b^	152/729 (21.0)	44/305 (14.4)	0.014 **
High cholesterol, HC(R)	166/748 (22.2)	48/320 (15.0)	0.007 **
High cholesterol, HC(F)	120/729 (16.5)	21/305 (6.9)	<0.001 **
Diabetes(R)	91/751 (12.1)	30/321 (9.3)	0.189
Diabetes (F)	62/729 (8.5)	14/305 (4.6)	0.028 **
Coronary heart disease, CHD(R)	20/749 (2.7)	6/321 (1.9)	0.521
Coronary heart disease, CHD(F)	20/728 (2.7)	2/304 (0.7)	0.034 **
Stroke(R)	10/750 (1.3)	3/321 (0.9)	0.585
Stroke (F)	16/729 (2.2)	3/305 (1.0)	0.186

^a^ disease of respondent (R) ^b^ disease of respondent’s family member (F) * P–value of chi square test for difference between diseases prevalence among biomass use and LPG use ** Significant at P < 0.05, 2-tailed test

Further analysis using logistic regression and control variables, revealed that compared to gas users, biomass users had family members with elevated CHD, hypertension, HC, and diabetes ( Table 4). Among different types of fuel, household cooks using wood had a significant elevated risk of CHD (OR=7.64, 95%CI 1.18-49.61), and their family members had an elevated risk of HC (OR=1.52, 95%CI 1.04-2.24). Comparing frequency of charcoal use, those who use charcoal sometimes or often are more likely to have CHD, hypertension, HC, and diabetes as compared to those who never use charcoal. The family members of charcoal users also had a significant increase of HC and stroke. When using TWC as an indicator for smoke exposure, it was found that those who always had TWC had significantly increased risk of stroke (OR=2.16; 95%CI 1.08-4.32), and those with sometimes TWC had a CHD risk (OR=2.64; 95%CI 1.02-6.81). Regarding kitchen location, the family members of cooks having kitchens both inside and outside a house had an elevated risk of stroke (OR=4.60; 95%CI 1.14-18.54).

**Table 4.** Association (OR) * between biomass use and cardiovascular outcomes.

	Hypertensio(R)	(F)	HC(R)	HC(F)	Diabetes(R)	Diabetes(F)	CHD(R)	CHD(F)	Stroke(R)	Stroke (F)
**Biomass use**										
Yes	1.27 (0.91–1.77)	**1.61 (1.10–2.35)**	1.28 (0.88–1.86)	**2.74 (1.66–4.53)**	1.08 (0.69–1.70)	**1.88 (1.03–3.46)**	1.18 (0.46–3.02)	4.35 (0.10–18.97	1.27 (0.33–4.87)	4.45 (0.97–20.56)
No	1.0	1.0	1.0	1.0	1.0	1.0	1.0	1.0	1.0	1.0
**Fuel type**										
Wood	1.11 (0.45–2.74)	1.93 (0.77–4.85)	1.31 (0.51–3.36)	1.45 (0.47–4.41)	0.58 (0.13–2.59)	2.11 (0.59–7.48)	1.63 (0.19–13.66)	NA	**7.64 (1.18–** **49.61)**	0.72 (0.27–1.96)
Charcoal	0.79 (0.57–1.09)	1.22 (0.86–1.72)	0.89 (0.63–1.26)	**1.52 (1.04–2.24)**	0.97 (0.63–1.48)	1.47 (0.89–2.43)	1.14 (0.49–2.65)	1.54 (0.64–3.73))	2.03 (0.58–7.09)	0.93 (0.68–1.26)
LPG/Electric	1.0	1.0	1.0	1.0	1.0	1.0	1.0	1.0	1.0	1.0
**Charcoal use**										
Often	1.10 (0.79–1.52)	1.10 (0.77–1.57)	1.30 (0.90–1.87)	**1.52 (1.02–2.27)**	1.44 (0.92–2.26)	1.56 (0.94–2.61)	1.33 (0.51–3.48)	1.37 (0.52–3.63)	1.66 (0.44–6.29)	**3.17 (1.04–9.71)**
Sometimes	**2.04 (1.32–3.15)**	1.28 (0.80–2.04)	**2.61 (1.63–4.18**)	1.11(0.63–1.96)	**2.09 (1.17–3.73)**	0.69 (0.29–1.61)	**4.11 (1.40–12.11)**	1.50 (0.44–5.18)	2.76 (0.56–13.50)	0.59 (0.07–5.21)
Never	1.0	1.0	1.0	1.0	1.0	1.0	1.0	1.0	1.0	1.0
**Tears while cooking**										
Often	0.84 (0.41–1.73)	0.71 (0.31–1.65)	0.56 (0.23–1.35)	0.76 (0.29–2.01)	0.93 (0.34–2.55)	0.23 (0.03–1.75)	3.45 (0.66–17.94)	1.07 (0.13–8.78)	**2.16 (1.08–4.32)**	1.80 (0.96–3.35)
Sometimes	0.82 (0.61–1.11)	0.96 (0.69–1.34)	0.92 (0.66–1.28)	1.02 (0.70–1.49)	1.17 (0.78–1.77)	0.80 (0.49–1.29)	**2.64 (1.02–6.81)**	1.13 (0.47–2.73)	0.98 (0.69–1.41)	1.04 (0.78–1.40)
Rarely	1.0	1.0	1.0	1.0	1.0	1.0	1.0	1.0	1.0	1.0
**Kitchen location**										
Inside	0.80 (0.58–1.11)	0.94 (0.67–1.34)	1.11 (0.78–1.58)	1.07 (0.72–1.60)	0.83 (0.54–1.27)	0.71 (0.42–1.18)	2.14 (0.77–5.92)	2.11 (0.75–5.93)	1.89 (0.45–7.89)	1.87 (0.55–6.33)
Both	1.00 (0.61–1.64)	1.08 (0.64–1.83)	0.85 (0.48–1.51)	0.84 (0.44–1.61)	1.17 (0.62–2.20)	0.76 (0.35–1.65)	1.96 (0.45–8.55)	0.50 (0.06–4.36)	4.62 (0.81–26.47)	**4.60 (1.14–18.54)**
Outside	1.0	1.0	1.0	1.0	1.0	1.0	1.0	1.0	1.0	1.0
**Charcoal use**										
Often	1.10 (0.79–1.52)	1.10 (0.77–1.57)	1.30 (0.90–1.87)	**1.52 (1.02–2.27)**	1.44 (0.92–2.26)	1.56 (0.94–2.61)	1.33 (0.51–3.48)	1.37 (0.52–3.63)	1.66 (0.44–6.29)	**3.17 (1.04–9.71)**
Sometimes	**2.04 (1.32–3.15)**	1.28 (0.80–2.04)	**2.61 (1.63–4.18**)	1.11(0.63–1.96)	**2.09 (1.17–3.73)**	0.69 (0.29–1.61)	**4.11 (1.40–12.11)**	1.50 (0.44–5.18)	2.76 (0.56–13.50)	0.59 (0.07–5.21)
Never	1.0	1.0	1.0	1.0	1.0	1.0	1.0	1.0	1.0	1.0
**Using charcoal location**										
Inside	0.95 (0.62–1.45)	0.89 (0.56–1.42)	1.26 (0.81–1.96)	1.40 (0.84–2.34)	0.72 (0.41–1.26)	0.83 (0.41–1.66)	2.13 (0.75–6.09)	**4.71 (1.36–16.32)**	1.32 (0.27–6.42)	2.46 (0.67–9.01)
Both	2.03 (0.89–4.63)	0.64 (0.21–1.92)	1.32 (0.54–3.25)	1.18 (0.38–3.64)	1.19 (0.42–3.38)	0.84 (0.19–3.78)	1.46 (0.16–13.01)	2.83 (0.30–27.07)	3.35 (0.24–47.21)	2.19 (0.22–21.51)
Outside	1.0	1.0	1.0	1.0	1.0	1.0	1.0	1.0	1.0	1.0
**Using charcoal duration (year)**										
>20	**1.38 (1.01–1.89)**	1.05 (0.74–1.50)	**1.73 (1.22–2.44)**	1.34 (0.89–2.01)	1.50 (0.98–2.30)	1.55 (0.93–2.57)	1.36 (0.99–1.87)	1.49 (0.60–3.74)	2.39 (0.70–8.24)	1.63 (0.51–5.19)
1–20	1.28 (0.78–2.12)	1.40 (0.87–2.25)	1.25 (0.70–2.22)	1.54 (0.89–2.68)	1.31 (0.65–2.63)	0.55 (0.21–1.46)	1.28 (0.77–2.11)	0.71 (0.15–3.46)	NA	2.62 (0.75–9.16)
Not use	1.0	1.0	1.0	1.0	1.0	1.0	1.0	1.0	1.0	1.0
Years of using charcoal o	1.003 (0.996–1.010)	1.003 (0.995–1.011)	**1.010** **(1.002–1.017)**	1.007 (0.998–1.016)	1.004 (0.995–1.013)	**1.013** **(1.001–1.024)**	1.015 (0.997–1.034)	1.012 (0.990–1.033)	1.014 (0.989–1.039)	1.011 (0.986–1.037)

*Logistic regression, adjusted for gender (male, female), age (continuous), cigarette smoking (ever, never), living with smoker (yes, no), working with smoker (yes, no), pesticide use (yes, no), working in a factory (yes, no) Significant value (p<0.05) is given in bold letters

## Discussion

This study presented an association between cardiovascular diseases and exposure to smoke from biomass, mainly charcoal, which is relatively cleaner when compared to wood, coal, or dung, a biomass which were often found in the literature. The study also showed that biomass use not only affects household cooks but also their family members. It was found that biomass users have a higher prevalence of hypertension and HC, and their family members had a higher prevalence of hypertension, HC, diabetes, and CHD ( Table 3). Further analysis using logistic regression and control for potential confounder showed a significant OR of biomass use and CHD(F), hypertension(F), HC(F), and diabetes(F) ( Table 4). Compared to LPG, wood use also had a strong association with stroke (OR=7.64; 95%CI 1.18–49.61). Among charcoal users, those who use it sometimes or often had an elevated risk of CHD, hypertension, HC, and diabetes for themselves, and risk of HC and stroke for their family members. The results are consistent with the literature. Previous research found biomass smoke contains a lot of pollutants, especially fine particulates, and carbon monoxide which are known to cause cardiovascular effects ^2^. In laboratory studies, biomass smoke exposure was associated with endothelial inflammation ^23^.

For hypertension, we found both cooks and their family members have a higher prevalence of hypertension ( Table 3). Further analysis indicated an elevated risk of hypertension (OR=1.61; 95%CI 1.10–2.35) among family members of cooks using biomass for cooking ( Table 4). As compared to those who never use it, cooks who sometimes use charcoal have twice the risk of hypertension (OR=2.04; 95%CI 1.32–3.15) and those who use charcoal over twenty years have 1.38 times the risk of hypertension (OR=1.38; 95%CI 1.01–1.89). In the literature, there is increasing evidence to link biomass smoke and hypertension ^25, 26^. A study in Peru found that biomass users had an increased risk of both prehypertension (OR=5.0; 95%CI 2.6–9.9), and hypertension (OR=3.5; 95%CI 1.7–7.0) ^13^. In Bangladesh, it was found that among rural women, one additional year of biomass smoke exposure to increase risk of hypertension by 61% (OR=1.61; 95%CI 1.16–2.22) ^14^. Recent studies in Honduras also linked PM2.5 and black carbon exposure and hypertension among women using traditional and improved stoves ^26^.

The current study also found a higher prevalence of HC among cooks and their family members using biomass fuel ( Table 3) with a significant OR of 2.74 (95%CI 1.66–4.53) for family members ( Table 4). The result showed a difference in the risk of HC among those who use wood, charcoal, and LPG. This risk also varied particularly according to the frequency of charcoal use. Compared to nonusers, an elevated risk of HC was found among cooks who sometimes use charcoal (OR=2.04; 95%CI 1.32–3.15), and among those who use charcoal over 20 years (OR=1.73; 95%CI 1.22–2.44). Among cooks, every year of using charcoal will increase risk of HC by about 1% (OR=1.010; 95%CI 1.002–1.017). Risk of HC was also increased among family members of cooks who often use charcoal (OR=1.52; 95%CI 1.02–2.27). Though the evidence was limited, other studies have found an association between cholesterol and COPD, a disease often found among biomass users ^3^. A study in Ghana also found a strong association between wood smoke exposure and several hematological and biochemical indices, including HC (OR=20.44; 95%CI 2.610–160.2) ^27^. The higher OR might be explained by the difference in biomass types, which was found to be wood in other studies, while most of respondents in this study use charcoal which is relatively cleaner.

We found about 10% of the respondents had type 2 diabetes and the prevalence of the disease was higher among biomass users ( Table 3). Logistic regression analysis revealed a significant risk of diabetes among cooks using charcoal sometimes (OR=2.09; 95%CI 1.17–3.73) as compared to the never user group ( Table 4). Among family members of cooks, risk of diabetes was elevated by using biomass fuel (OR=1.88; 95%CI 1.03–3.46), and years of using charcoal (OR=1.013; 95%CI 1.001–1.024). Similar results have also been reported by several studies on the effect of particulate matter or traffic-related air pollutants on diabetes ^28^. In addition, experimental studies may provide potential mechanisms, including glucose homeostasis, systemic inflammation, stress in the liver and endoplasmic reticulum, and alterations of mitochondrial and other adipose tissue ^29^. Currently, epidemiological studies on the effect of indoor air pollution on diabetes are rare. A study of women in Honduras reported an association between the prevalence of prediabetes/diabetes and PM2.5 in kitchen biomass cooking stoves ^30^. This was consistent with the results from a previous study from Shanghai Putuo, which also found an elevated risk of several cardiovascular diseases including diabetes (OR=2.48; 95%CI 1.59–3.86) among people using solid fuel at home ^15^.

Those who use biomass for cooking had a risk of CHD 4.35 times (95%CI 0.10–18.97) of LPG users; and those using charcoal sometimes had risk of CHD 4.11 times (95%CI 1.40–12.11) of never user group. These results are consistent with evidence from cigarette smoke and ambient air pollution. In animal studies, biomass fuel smoke caused arteriosclerotic effects in animal blood vessels ^12^. Studies found COPD as a risk factor of CHD ^31^; and our previous study found elevated chronic symptoms, such as chronic cough, dyspnea and runny nose which is a sign of COPD among cooks using biomass fuel for cooking ^3^. Epidemiological studies also reported an association between solid fuel smoke exposure and CHD ^32^. A study in Pakistan found that rural women who currently use solid fuel had an increased risk of acute coronary syndrome (OR=4.8; 95%CI 1.5–14.8) ^33^. This is consistent with a study from Shanghai Putuo, which found solid fuel use in the home is associated with CHD (OR=2.58; 95%CI 1.53–4.32) ^15^, and study from Shanxi, China found an elevated risk of CHD (OR=2.25) among solid fuel users ^16^.

In this study, respondents who use wood (OR=7.64; 95%CI 1.18–49.61) and charcoal (OR=2.03; 95%CI 0.58–7.09) had an elevated risk of stroke as compared to clean fuel users ( Table 4). Among charcoal users, those using charcoal sometimes (OR=1.66; 95%CI 0.44–6.29) and often (OR=2.76; 95%CI 0.56–13.50) seem to have a higher risk of stroke but a significant elevation was found only among the family members of cooks using charcoal often (OR=3.17; 95%CI 1.04–9.71). This was consistent with the literature. The association between household solid fuel use and stoke were also reported in a study from Shanghai Putuo (OR=1.87; 95% CI 1.03–3.38) ^15^, and study from Shanxi, China (OR=1.64) ^16^. In ambient settings, a long-term effect of PM exposure on cardiovascular disease, including stroke, was well established ^34^. It was estimated that for each 10 µg/m ^3^ increment in PM10, risk of overall stroke events will increase by 1.06 times (95%CI 1.02–1.11), and the risk of stoke mortality by 1.08 times (95%CI 0.99–1.18) ^35^.

One potential drawback of this study was the use of self-reported data of diseases. Without the confirmation of medical records, the survey diseases are subjected to information bias. However, the bias will be distributed equally to all comparison groups, and this tends to underestimate the result. The number of participants included in this study was also rather small to detect the actual association of a rare disease, e.g. stroke. By using cross-sectional design, the study result cannot explain the causal relationship, because it is not known whether exposure or the disease occurred first. However, the problem is minimal for rare diseases.

## Conclusions

The results from this study support research findings in other part of the world that using biomass for cooking increases the risk of cardiovascular diseases. This study also confirms the negative effects of using charcoal, which is considered to be a relatively cleaner fuel as compared with wood, dung, coal, and other agricultural residues. Concerned organizations should pay more attention to the issue and promote clean fuel usage.

## Data availability

### Underlying data

Figshare: Household cooking and cardiovascular diseases, https://doi.org/10.6084/m9.figshare.12117066.v2 ^23^.

This project contains the following underlying data:

- Household cooking and cardiovascular diseases.sav (Collected demographic and cardiovascular diseases data)
- Data dictionary.docx (Word document containing dictionary for study dataset)

### Extended data

Figshare: Questionnaire-household cooking and cardiovascular disease, https://doi.org/10.6084/m9.figshare.12121887.v2 ^22^.

This project contains the following extended data:

- Questionnaire-household cooking and cardiovascular disease.docx (Study questionnaire in English)
- Questionnaire-household cooking and cardiovascular disease-Thai.docx (Study questionnaire in Thai)

Data are available under the terms of the Creative Commons Zero "No rights reserved" data waiver (CC0 1.0 Public domain dedication).

## References

[1] WHO: Household Energy and Health Household Energy and Health.Geneva, Switzerland;2006.

[2] Naeher LP Brauer M Lipsett M: Woodsmoke health effects: a review. *Inhal Toxicol.* 2007;19(1):67–106. 10.1080/08958370600985875 17127644

[3] Juntarawijit Y Juntarawijit C: Cooking smoke exposure and respiratory symptoms among those responsible for household cooking: A study in Phitsanulok, Thailand. *Heliyon.* 2019;5(5):e01706. 10.1016/j.heliyon.2019.e01706 31193378 PMC6526227

[4] Kim KH Jahan SA Kabir E: A review of diseases associated with household air pollution due to the use of biomass fuels. *J Hazard Mater.*Elsevier;2011;192(2):425–31. 10.1016/j.jhazmat.2011.05.087 21705140

[5] Fullerton DG Bruce N Gordon SB: Indoor air pollution from biomass fuel smoke is a major health concern in the developing world. *Trans R Soc Trop Med Hyg.*Elsevier;2008;102(9):843–51. 10.1016/j.trstmh.2008.05.028 18639310 PMC2568866

[6] Owili PO Muga MA Pan WC: Cooking fuel and risk of under-five mortality in 23 Sub-Saharan African countries: a population-based study. *Int J Environ Health Res.* 2017;27(3):191–204. 10.1080/09603123.2017.1332347 28552005

[7] WHO: Indoor air pollution.2020 Reference Source

[8] Haber G Witberg G Danenberg H: [Air pollution and cardiovascular disease]. *Harefuah.* 2007;146(10):738–43. 17990383

[9] Mortimer K Gordon SB Jindal SK: Household air pollution is a major avoidable risk factor for cardiorespiratory disease. *Chest.*American College of Chest Physicians;2012;142(5):1308–15. 10.1378/chest.12-1596 23131939 PMC5991547

[10] Rajagopalan S Brook RD: THE INDOOR-OUTDOOR AIR-POLLUTION CONTINUUM AND THE BURDEN OF CARDIOVASCULAR DISEASE: AN OPPORTUNITY FOR IMPROVING GLOBAL HEALTH. *Glob Heart.*Elsevier;2012;7(3):207–13. 10.1016/j.gheart.2012.06.009 23181202 PMC3501678

[11] Burroughs Peña MS Velazquez EJ Rivera JD: Biomass fuel smoke exposure was associated with adverse cardiac remodeling and left ventricular dysfunction in Peru. *Indoor Air.* 2017;27(4):737–45. 10.1111/ina.12362 27990700 PMC5489120

[12] Painschab MS Davila-Roman VG Gilman RH: Chronic exposure to biomass fuel is associated with increased carotid artery intima-media thickness and a higher prevalence of atherosclerotic plaque. *Heart.* 2013;99(14):984–91. 10.1136/heartjnl-2012-303440 23619984 PMC4657551

[13] Burroughs Peña M Romero KM Velazquez EJ: Relationship between daily exposure to biomass fuel smoke and blood pressure in high-altitude Peru. *Hypertension.* 2015;65(5):1134–40. 10.1161/HYPERTENSIONAHA.114.04840 25753976 PMC4466100

[14] Barman N Haque MA Rahman AKMF: Association of biomass fuel smoke exposure and hypertension among rural women of Bangladesh: A cross-sectional study. *Indian J Public Health.* 2019;63(3):258–60. 10.4103/ijph.IJPH_462_18 31552859

[15] Lee MS Hang JQ Zhang FY: In-home solid fuel use and cardiovascular disease: a cross-sectional analysis of the Shanghai Putuo study. *Environ Health.* 2012;11(1): 18. 10.1186/1476-069X-11-18 22455369 PMC3349503

[16] Qu W Yan Z Qu G: Household Solid Fuel Use and Cardiovascular Disease in Rural Areas in Shanxi, China. *Iran J Public Health.* 2015;44(5):625–38. 26284203 PMC4537619

[17] Yu K Lv J Qiu G: Cooking fuels and risk of all-cause and cardiopulmonary mortality in urban China: a prospective cohort study. *Lancet Glob Health.* 2020;8(3):e430–9. 10.1016/S2214-109X(19)30525-X 31972151 PMC7031698

[18] World Health Organization: Cardiovascular diseases (CVDs).Fact Sheets. 2017. Reference Source

[19] World Health Organization: Noncommunicable Disease (NCD) Country Porofiles, 2018.2018 Reference Source

[20] NSO National Office of Statitics, UNICEF, Fund UNC, *et al.*: Thailand Thailand Monitoring the situation of children and women Multiple Indicator Cluster Survey.2012.

[21] Wikipedia: Phitsanulok Province.2020 Reference Source

[22] Juntarawijit C: Questionnaire-household cooking and cardiovascular diseases. *figshare.*Dataset.2020 10.6084/m9.figshare.12121887.v2

[23] Juntarawijit C: Household cooking and cardiovascular diseases. *figshare.*Dataset.2020 10.6084/m9.figshare.12117066.v2

[24] Caravedo MA Herrera PM Mongilardi N: Chronic exposure to biomass fuel smoke and markers of endothelial inflammation. *Indoor Air.* 2016;26(5):768–75. 10.1111/ina.12259 26476302 PMC4935667

[25] Dutta A Ray MR: Hypertension and respiratory health in biomass smoke-exposed premenopausal Indian women. *Air Qual Atmos Heal.* 2014;7(2):229–38. 10.1007/s11869-013-0228-5

[26] Young BN Clark ML Rajkumar S: Exposure to household air pollution from biomass cookstoves and blood pressure among women in rural Honduras: A cross-sectional study. *Indoor Air.* 2019;29(1):130–42. 10.1111/ina.12507 30195255 PMC6301093

[27] Dadzie EK Ephraim RKD Afrifa J: Persistent exposure to wood smoke is associated with variations in biochemical and hematological indices among regular wood burners in the Cape Coast metropolis, Ghana. *Sci African.* 2019;4:e00100 10.1016/j.sciaf.2019.e00100

[28] Park SK: Ambient air pollution and type 2 diabetes: Do the metabolic effects of air pollution start early in life? *Diabetes.*American Diabetes Association Inc;2017;66(7):1755–7. 10.2337/dbi17-0012 28637828 PMC5482079

[29] Rajagopalan S Brook RD: Air pollution and type 2 diabetes: mechanistic insights. *Diabetes.*American Diabetes Association;2012;61(12):3037–45. 10.2337/db12-0190 23172950 PMC3501850

[30] Rajkumar S Clark ML Young BN: Exposure to household air pollution from biomass-burning cookstoves and HbA1c and diabetic status among Honduran women. *Indoor Air.* 2018;28(5):768–76. 10.1111/ina.12484 29896912 PMC6292747

[31] Müllerova H Agusti A Erqou S: Cardiovascular comorbidity in COPD: systematic literature review. *Chest.* 2013;144(4):1163–78. 10.1378/chest.12-2847 23722528

[32] Fatmi Z Coggon D: Coronary heart disease and household air pollution from use of solid fuel: a systematic review. *Br Med Bull.* 2016;118(1):91–109. 10.1093/bmb/ldw015 27151956 PMC4973663

[33] Fatmi Z Coggon D Kazi A: Solid fuel use is a major risk factor for acute coronary syndromes among rural women: a matched case control study. *Public Health.* 2014;128(1):77–82. 10.1016/j.puhe.2013.09.005 24342134 PMC3964605

[34] Lee KK Miller MR Shah ASV: Air pollution and stroke. *J Stroke.*Korean Stroke Society;2018;20(1):2–11. 10.5853/jos.2017.02894 29402072 PMC5836577

[35] Scheers H Jacobs L Casas L: Long-Term Exposure to Particulate Matter Air Pollution Is a Risk Factor for Stroke: Meta-Analytical Evidence. *Stroke.* 2015;46(11):3058–66. 10.1161/STROKEAHA.115.009913 26463695

