# Supplementary material for: Cooking with biomass fuel and cardiovascular disease: a cross-sectional study among rural villagers in Phitsanulok, Thailand
Source: F1000Res. 2020 Oct 8;9:307. Originally published 2020 Apr 29. [Version 2] doi: 10.12688/f1000research.23457.2 (PMC7549177; doi:10.12688/f1000research.23457.2)
Supplement: Peer review history

# Peer review: Reviewer response for version 2

**DOI:** 10.5256/f1000research.29890.r72717

**Published:** 2020-11-05

**Authors:** Ogonna N.O. Nwankwo

**Affiliations:** 1 Department of Community Medicine, University of Calabar Teaching Hospital, Calabar, Nigeria

First off, thank you for setting out to provide more evidence on this important public health issue that affects a large proportion of people globally.

 I will categorize my comments under major and minor issues with the manuscripts that I believe should be worked on.

**Major issues:**

 I believe there is a need for reanalysis of the data based on the issue I have raised in the result section concerning the definition of the biomass use which is a major variable of interest for this study i.e. classification of participants into biomass users and non-biomass users. This may probably result in a significant change in the results outputs, discussion and conclusion of your study.

**Minor issues:**

 I am going to make my feedback based on the structure of the manuscript. Some of my comments have been made by the other peer reviewers but seems to have been missed in this most current version.

**Abstract:**

- In the abstract, it may be good to write in full LPG. I believe it is best to use standardized abbreviations. I am not sure that HC is a widely used abbreviation?
- *“The **object** of this cross-sectional study was …….” *

           Do you mean “The **objective **of this cross-sectional study was …….”?

  

- *“In each household, data on cardiovascular disease, cooking practices, and cooking fuel, types of fuel they normally used for cooking, were collected.”*

          Can be better phrased as: "In each household, data on cardiovascular disease, cooking practices, cooking fuel and types of fuel they normally used for cooking were collected."

  

- *“…and supports that exposure to biomass smoke increase cardiovascular diseases.”*

      Do you mean “…and supports that exposure to biomass smoke **increases the risk of **cardiovascular diseases.”?

**Introduction:**

- “ *In addition to fuel burning smoke, overheated of cooking oils might also produce smoke which depended on several factors, including cooking oils, cooking methods, cooking equipment, and food types3”*

        This sentence needs to be rephrased to ensure better clarity.

  

- *"non-respiratory illness, such as poor lung function, low birthweight, **nutritionaldeficiency**, and impairment of learning ability6,7"*
  Space the bolded word as: "non-respiratory illness, such as poor lung function, low birthweight, **nutritional deficiency**, and impairment of learning ability6,7".
- *“….still use biomass, mainly charcoal, wood, and agriculture residue, for cooking” *
  Can better read as: “….still use biomass mainly charcoal, wood and agriculture residue for cooking”.
- *“..between biomass use for cooking and cardiovascular diseases, including CHD, hypertension, HC, diabetes, and stroke.”*
  Can better read as: “...between biomass use for cooking and cardiovascular diseases including CHD, hypertension, HC, diabetes and stroke.”
- *‘The result could be used for disease prevention and control, and to support the global literature.”*
  Can you better expatiate on this statement?

**Methods:**

  

- *“This is cross-sectional study.”*
  Can better read as: “This is **a** cross-sectional study”.
- I observed that the questionnaire was translated. What was the process used in the translation and maintaining its validity? I believe you need to address this.

- *“Ever smoker referred to those who smoke more than 100 cigarettes in their lifetime.”*
  Can better read as: *“Ever smoker referred to those who have smoked more than 100 cigarettes in their lifetime.”*
- *“….Living smoker..” *
  “Living with smoker”

- **Under the statistical analysis, could you add the explanation of how you derived your outcome variable of biomass and non-biomass use from the questionnaire?**

**Results:**

  

- *“The highest education levels were primary school or high school.”*

        Rephrase this sentence.

  

- *“About 70% of the respondents reported using biomass for cooking ( **Table 2**). However, when asked for fuel types that they usually use for cooking, 64.5% reported LPG and 32.3% charcoal. Among those who use charcoal, 38.6% use it often.”*

**Reading your response to one of the earlier peer reviewers, I would suggest you reanalyze your data using the participants' response of their fuel use type and further reclassify the different types of fuel types into biomass (wood, charcoal) and non-biomass user (LPG, electricity). From your questionnaire it appears question 16 and 17 should be from where you should derive the answer.**

**I expect that people are more likely to know what fuel type they use but may not be adequately informed to know technical terms like biomass fuel and non-biomass fuel.**

**All in all you need to be extremely clear on your definition of biomass use and this definition should be added to the method section as I believe some people may have primary and secondary use of fuel type of which Q17 from your questionnaire tries to address.**

****

  

- Looking at this statement and the table it appears that over 65.4% of the participants are non-biomass users i.e. users of electricity and LPG. Please can you clarify how you derived the opposite i.e. that biomass users are 70%. This does not appear to tally with the figures on the table.
  Again the data is found in Table 1 and not Table 2, except you rename all your tables.
- Can you also clarify why “never users of charcoal” under the Frequency of using charcoal (495) is different from not use group under duration of use of charcoal (502).
- **It appears you may need to refine the headings to be more self–explanatory.**
- Under table 2: can the headings include the number of participants under each category e.g. Biomass, n (%) N=756.
- Be consistent in using one term for people not using biomass as in some places you used non-user group and in some others, gas users. Meanwhile did you aggregate electricity users and LPG users? If so you may need to state it.
- *“..and their family members also had more **incidence** of hypertension, HC, diabetes, and heart disease.”*

Please replace as this study cannot assess for incidence but for prevalence.

- “..and their family members also had more prevalence of hypertension, HC, diabetes, and heart disease.”
- In Table 4 put a superscript at R and F to explain what it is like in Table 3. It would have been good to label the continuation of Table 4.
- Is there any reason why you did not control for occupation and education in the logistic regression? Or don’t you think they potentially can influence the results?

**Discussion:**

- *“This study presented an association between cardiovascular diseases and exposure to smoke from biomass, **mainly charcoal**, which is relatively cleaner when compared to wood,* *coal, or dung, a biomass which were often found in the literature.”*
  If you planned to concentrate on charcoal then you should have clearly stated it and brought it out even in the result sections. It appears you may not have adequately brought this out in the write up and result so far. Again, any reference to show the statement you made that charcoal is cleaner than the others? I believe that is a statement that will need to be backed up with evidence and also should have been in the introduction.
- *Further analysis using logistic regression and control for potential confounder showed a significant OR of biomass use and **CHD(F), hypertension(F), HC(F), and diabetes(F)***
  I think it may be good to write the bolded in full.
- **The mentioned high cholesterol (HC) is instead a biochemical abnormality and not a disease condition.**
- I believe this is a major comment, you may need to address as it may cast a doubt on the findings related to this given variable as we would expect most people not to know their HC number except they have all been tested for it unlike being diagnosed of a disease.
- So is HC routinely carried out for the villagers? If yes by whom and are they told their results? HC is a risk factor for some of the NCDs.
- **If not I would suggest that this is deleted or it is clearly highlighted as doubtful.**

Is the work clearly and accurately presented and does it cite the current literature?

Yes

If applicable, is the statistical analysis and its interpretation appropriate?

No

Are all the source data underlying the results available to ensure full reproducibility?

Yes

Is the study design appropriate and is the work technically sound?

Partly

Are the conclusions drawn adequately supported by the results?

Yes

Are sufficient details of methods and analysis provided to allow replication by others?

Partly

Reviewer Expertise:

Public health, Epidemiology and Biostatistics, Health policy and health system research, Chronic diseases.

I confirm that I have read this submission and believe that I have an appropriate level of expertise to confirm that it is of an acceptable scientific standard, however I have significant reservations, as outlined above.

# Peer review: Reviewer response for version 2

**DOI:** 10.5256/f1000research.29890.r72681

**Published:** 2020-10-09

**Authors:** Nilima Barman

**Affiliations:** 1 Department of Laboratory Medicine, Bangladesh Institute of Research and Rehabilitation for Diabetes, Endocrine and Metabolic Disorders (BIRDEM), Dhaka, Bangladesh

Now the article is more precise. The authors has addressed most of the review points from their point of view with clear justification.

Is the work clearly and accurately presented and does it cite the current literature?

Yes

If applicable, is the statistical analysis and its interpretation appropriate?

Partly

Are all the source data underlying the results available to ensure full reproducibility?

Yes

Is the study design appropriate and is the work technically sound?

Partly

Are the conclusions drawn adequately supported by the results?

Yes

Are sufficient details of methods and analysis provided to allow replication by others?

Partly

Reviewer Expertise:

Non communicable disease, Laboratory medicine, Public health

I confirm that I have read this submission and believe that I have an appropriate level of expertise to confirm that it is of an acceptable scientific standard.

# Peer review: Reviewer response for version 1

**DOI:** 10.5256/f1000research.25888.r71210

**Published:** 2020-09-22

**Authors:** Nilima Barman

**Affiliations:** 1 Department of Laboratory Medicine, Bangladesh Institute of Research and Rehabilitation for Diabetes, Endocrine and Metabolic Disorders (BIRDEM), Dhaka, Bangladesh

The article has emphasized on detrimental effects of biomass cooking fuel on cardiovascular health. The results will help in future policy making regarding cooking fuel. The overall article is well-written but some of the issues need to be clarified as described below.

**In the abstract: **

1. The methods should be improved, giving a clear description of cooking fuel.
2. The author mentioned 'hypertension' in the objective, but high blood pressure (HBP) in the result section. Terminology should be consistent throughout the manuscript.
3. In the conclusion, the author stated, 'the study results were consistent with those found in studies from other parts of the world...'. This statement should be discussed in the discussion, not in the conclusion. The conclusion should be based on the authors' main findings.

**In the introduction:**

1. Please clarify the term 'Cooking smoke,' whether it means smoke from cooking or from fuel?

**In the methods:**

1. The study design is well articulated. But self-reported cardiovascular diseases may give a vague impression to the readers, although the author mentioned it as a limitation. In a matter of sense, the authors showed more than two-thirds (71.9 %) of participants had primary education who acted as self reporters of disease condition. So, in my opinion, the authors should have a strong justification in favor of including self-reported cardiovascular diseases with authentic scientific references.
2. Again, do the authors cross-check self-reported disease conditions with the patient's medical or laboratory reports or drug history? The mentioned high cholesterol (HC) is instead a biochemical abnormality apart from a disease condition.

**In statistical analysis:**

1. The regression model needs a precise description. Is it a multivariate or multinominal model?
2. In a logistic regression model, the cardiovascular disease condition of family members are also encountered. Are the adjusting confounding variables like age and sex in that regression model in relation of family members’ age and sex, or the respondents’? It should be precisely mentioned in description of regression model.

**In results:**

1. It seems confusing between the data on 'Fuel use for cooking' in Table 1 and the total no of biomass and LPG users in Table 2. (In Table 1, LPG users are 695 participants, but in Table 2, it is 322 (by addition of 56 male and 266 female). Same for the biomass fuel. Please clarify it.
2. The footnote of Table 2 had a term '2-trail'. Is it trail or tail? Please correct it.
3. In Table 4, the significant value is given in bold letters. Please mention it.

**In discussion:**

1. In the 4 ^th^ paragraph, the authors stated that 'We found about 10% of the respondents had type 2 diabetes'. It is hard to believe self-reported evidence of type 2 diabetes. Please clarify this or correct it.     
               

**In conclusion:** the same as in abstract.

Is the work clearly and accurately presented and does it cite the current literature?

Yes

If applicable, is the statistical analysis and its interpretation appropriate?

Partly

Are all the source data underlying the results available to ensure full reproducibility?

Yes

Is the study design appropriate and is the work technically sound?

Partly

Are the conclusions drawn adequately supported by the results?

Yes

Are sufficient details of methods and analysis provided to allow replication by others?

Partly

Reviewer Expertise:

Non communicable disease, Laboratory medicine, Public health

I confirm that I have read this submission and believe that I have an appropriate level of expertise to confirm that it is of an acceptable scientific standard, however I have significant reservations, as outlined above.

# Response

**Published:** 2020-09-25

**Authors:** Chudchawal Juntarawijit

**Affiliations:** Naresuan University, Thailand

**Response to reviewer**

**In the abstract:**

Comments: The methods should be improved, giving a clear description of cooking fuel.

**Responses:** A short description of cooking fuel was added to the methods section. Because F1000Research has set a maximum limit of 300 words for abstract, no more detailed information could be added.

Comments: The author mentioned 'hypertension' in the objective, but high blood pressure (HBP) in the result section. Terminology should be consistent throughout the manuscript. 

**Responses:** The term “high blood pressure” was replaced by “hypertension”.

Comments: In the conclusion, the author stated, 'the study results were consistent with those found in studies from other parts of the world...'. This statement should be discussed in the discussion, not in the conclusion. The conclusion should be based on the authors' main findings. 

**Responses:** Yes, I agree that “the statement should be discussed in the discussion”. However, we believed we had already done that enough to justify the statement, which is our main finding.

**In the introduction:**

Comments: Please clarify the term 'Cooking smoke,' whether it means smoke from cooking or from fuel? 

**Responses:** The meaning of cooking smoke was clarified and more information was added to the first paragraph in Introduction.

**In the methods:**

Comments: The study design is well articulated. But self-reported cardiovascular diseases may give a vague impression to the readers, although the author mentioned it as a limitation. In a matter of sense, the authors showed more than two-thirds (71.9 %) of participants had primary education who acted as self reporters of disease condition. So, in my opinion, the authors should have a strong justification in favor of including self-reported cardiovascular diseases with authentic scientific references.

**Responses:**  Yes, I agree that using self-report data is a limitation of this study. However, since the data was collected by a well trained and experienced village health volunteer, the problem was expected to be minimal. The quality of the answer to this question may not depend much on their background education of respondents. In addition, this information bias, if occurred, will equally distribute among groups (case and control).  

Comments: Again, do the authors cross-check self-reported disease conditions with the patient's medical or laboratory reports or drug history? The mentioned high cholesterol (HC) is instead a biochemical abnormality apart from a disease condition.

**Responses:** Yes, it is good if we can do the cross-check self-reported conditions. However, we did not do that.

**In statistical analysis:**

Comments: The regression model needs a precise description. Is it a multivariate or multinominal model?

**Responses:** Thank you for reminding.

In this study, we use binary multiple logistic regression.

More detail of the model was added to the statistic description.

Comments: In a logistic regression model, the cardiovascular disease condition of family members are also encountered. Are the adjusting confounding variables like age and sex in that regression model in relation of family members’ age and sex, or the respondents’? It should be precisely mentioned in description of regression model.

**Responses:** Thank you to raise the issue. It is a good point which could be another limitation of this study. We didn’t have enough information of the family members, so just use the data of the respondents.

More information was added in the description of the regression model.

**In results:**

Comments: It seems confusing between the data on 'Fuel use for cooking' in Table 1 and the total no of biomass and LPG users in Table 2. (In Table 1, LPG users are 695 participants, but in Table 2, it is 322 (by addition of 56 male and 266 female). Same for the biomass fuel. Please clarify it.

**Responses: **Table 1 showed data on what types of fuel the respondents usually use for cooking, and some of them use more than one fuel types. However, data in Table 2 was from another question which asked whether the respondents use biomass or not, and those who answered “no” was then classed as none biomass user or LPG user (assumed that only few use electric strove).

Comments: The footnote of Table 2 had a term '2-trail'. Is it trail or tail? Please correct it.

**Responses:** The error was corrected.

Comments: In Table 4, the significant value is given in bold letters. Please mention it.

**Responses: **The statement was added to Table 4 footnote.

**In discussion:**

Comments:  In the 4 ^th^ paragraph, the authors stated that 'We found about 10% of the respondents had type 2 diabetes'. It is hard to believe self-reported evidence of type 2 diabetes. Please clarify this or correct it.     

**Responses:** Actually, we asked whether the respondents had ever been diagnosed by a medical doctor to have type 2 diabetes. Also the data was collected by village health volunteer, who were well trained as public health staff and know the disease which is very common in Thailand.

# Peer review: Reviewer response for version 1

**DOI:** 10.5256/f1000research.25888.r62897

**Published:** 2020-05-19

**Authors:** Thandi Puoane, Lungiswa Tsolekile

**Affiliations:** 1 School of Public Health, University of the Western Cape, Bellville, South Africa

A cross-sectional study undertaken to investigate a possible association between biomass use for cooking and cardiovascular diseases, including CHD, HBP, HC, diabetes, and stroke. Biomass users had a significantly higher prevalence of HBP, and HC, and their family members also had more incidence of HBP, HC, diabetes, and heart disease.

- In the abstract: Change "object" to "objective" of this study.
- The abbreviation “LPG" used from the abstract was not defined.
- The conclusion should be improved giving a summary of the findings first then move onto saying “these findings support those of..."

Is the work clearly and accurately presented and does it cite the current literature?

Yes

If applicable, is the statistical analysis and its interpretation appropriate?

I cannot comment. A qualified statistician is required.

Are all the source data underlying the results available to ensure full reproducibility?

Yes

Is the study design appropriate and is the work technically sound?

Yes

Are the conclusions drawn adequately supported by the results?

Yes

Are sufficient details of methods and analysis provided to allow replication by others?

Yes

Reviewer Expertise:

Public Health, Nutrition Epidemiology, Cardiovascular risk factors

We confirm that we have read this submission and believe that we have an appropriate level of expertise to confirm that it is of an acceptable scientific standard.
